# Comparison of Cerebellar Grey Matter Alterations in Bipolar and Cerebellar Patients: Evidence from Voxel-Based Analysis

**DOI:** 10.3390/ijms22073511

**Published:** 2021-03-29

**Authors:** Michela Lupo, Giusy Olivito, Andrea Gragnani, Marco Saettoni, Libera Siciliano, Corinna Pancheri, Matteo Panfili, Marco Bozzali, Roberto Delle Chiaie, Maria Leggio

**Affiliations:** 1 Ataxia Laboratory, Fondazione Santa Lucia IRCCS, 00179 Rome, Italy; g.olivito@hsantalucia.it (G.O.); maria.leggio@uniroma1.it (M.L.); 2 Department of Psychology, Sapienza University of Rome, 00185 Rome, Italy; 3 Scuola di Psicoterapia Cognitiva SPC, 58100 Grosseto, Italy; gragnani@apc.it (A.G.); marcosaettoni@gmail.com (M.S.); 4 Associazione Psicologia Cognitiva (APC)/Scuola di Psicoterapia Cognitiva (SPC), 00185 Rome, Italy; 5 Unità Funzionale Salute Mentale Adulti ASL Toscana Nord-Ovest Valle del Serchio, 56121 Pisa, Italy; 6 PhD Program in Behavioral Neuroscience, Sapienza University of Rome, 00185 Rome, Italy; libera.siciliano@uniroma1.it; 7 Departement of Neuroscience and Mental Health–Policlinico Umberto I Hospital, Sapienza University of Rome, 00161 Rome, Italy; corinnapancheri@hotmail.it (C.P.); matteopanfili93@gmail.com (M.P.); r.dellechiaie@centrokahlbaum.it (R.D.C.); 8 Clinical Imaging Science Center, Brighton and Sussex Medical School, Brighton BN1 9RR, UK; m.bozzali@hsantalucia.it

**Keywords:** cerebellar atrophy, bipolar disorder, voxel-based morphometry, cerebellar grey matter volume

## Abstract

The aim of this study was to compare the patterns of cerebellar alterations associated with bipolar disease with those induced by the presence of cerebellar neurodegenerative pathologies to clarify the potential cerebellar contribution to bipolar affective disturbance. Twenty-nine patients affected by bipolar disorder, 32 subjects affected by cerebellar neurodegenerative pathologies, and 37 age-matched healthy subjects underwent a 3T MRI protocol. A voxel-based morphometry analysis was used to show similarities and differences in cerebellar grey matter (GM) loss between the groups. We found a pattern of GM cerebellar alterations in both bipolar and cerebellar groups that involved the anterior and posterior cerebellar regions (*p* = 0.05). The direct comparison between bipolar and cerebellar patients demonstrated a significant difference in GM loss in cerebellar neurodegenerative patients in the bilateral anterior and posterior motor cerebellar regions, such as lobules I−IV, V, VI, VIIIa, VIIIb, IX, VIIb and vermis VI, while a pattern of overlapping GM loss was evident in right lobule V, right crus I and bilateral crus II. Our findings showed, for the first time, common and different alteration patterns of specific cerebellar lobules in bipolar and neurodegenerative cerebellar patients, which allowed us to hypothesize a cerebellar role in the cognitive and mood dysregulation symptoms that characterize bipolar disorder.

## 1. Introduction

Bipolar disorder (BD) is a severe and chronic psychiatric disease that is often associated with several medical conditions, including cardiovascular problems, diabetes mellitus, and neurovascular disease [1,2]. This disorder is characterized by episodes of mania (or hypomania) and depression that can lead to cognitive impairments with a considerable impact on one’s quality of life [1,3,4,5,6,7]. Cognitive impairments, mainly involving executive function, attention, verbal and episodic memory, persist during euthymic phases [8,9,10].

Furthermore, neuroimaging studies have shown that bipolar disorder is characterized by several structural modifications in the cortical and subcortical areas, such as grey matter volume abnormalities in the frontal and temporal regions and in limbic regions, such as the cingulate cortex [11,12,13]. Among subcortical structures, specific structural alterations have also been reported in the cerebellum.

Indeed, recent literature has shown cerebellar involvement in psychiatric diseases and neurodevelopmental disorders, specifically in schizophrenia, autism and obsessive-compulsive disorder [14,15,16,17,18,19,20,21]. Furthermore, in recent years, some evidence has highlighted the potential role of the cerebellum in other psychiatric conditions characterized by mood swings, such as bipolar disorder [22,23]. It has been shown that alterations of the cortico-cerebellar network in patients with bipolar disorder (type 1 and type 2) are present during earlier stages of the disease and remain stable over time, thus suggesting a possible neurodevelopmental involvement of this network in the mechanism of bipolar disorder, with no differences between bipolar disorder subtypes [23]. Despite this evidence, the specific cerebellar contribution to the neuropathophysiological mechanisms underlying bipolar disorder still needs to be clarified.

Indeed, neuroimaging studies have described cerebellar alterations in patients with bipolar disorder [24,25,26,27] over the last twenty years and researchers have focused on this structure [28,29,30,31,32] in light of the cerebellar connections with cortical areas involved in the pathophysiology of bipolar disorder [13,28,31,33,34] and the cerebellar role in emotion [35,36,37,38,39], social cognition [16,40,41] and cognitive functions [42,43,44,45]. Moreover, it is worth noting that very recently the onset of mood disorders (manic and depressive symptoms) has been demonstrated in the presence of isolated cerebellar lesions and neurodegenerative cerebellar pathologies [46,47,48,49]. These data confirm that cerebellar alterations are associated with mood symptoms, as reported in cerebellar-cognitive affective syndrome (CCAS) [50].

In light of these observations, it is reasonable to hypothesize that the cerebellar alteration in BD may contribute to specific features of bipolar symptoms (i.e., mania, hypomania, depression) and further insight can be gained from the comparison with neurodegenerative disorders that selectively affect the cerebellar cortex, such as spinocerebellar ataxia (SCA). To this aim, in the present study, cerebellar structural patterns were compared between BD patients and patients affected by cerebellar degenerative disorders (CD) of different etiology. Indeed, according to the growing evidence of a cerebellar involvement in the manic symptoms of bipolar disorder and the presence of mood disturbance in cerebellar diseases, the comparison between BD and CD patients will provide a crucial insight into understanding the cerebellum role in the pathophysiology of mood disturbances and in maintaining symptoms related to bipolar disorder.

## 2. Results

All CD patients enrolled in the present study obtained a normal Intelligence Quotient (IQ > 70). There were no differences in terms of age (F = 0.995; *p* = 0.374) or sex distribution (F = 0.042; *p* = 0.959) between BD, CD and healthy subjects (HS), as assessed by one-way ANOVA (Table 1).

As assessed by the international cooperative ataxia rating scale (ICARS), neurological examination revealed the presence of a pure cerebellar motor syndrome in CD. No extracerebellar symptoms were present at the time of the study. Moreover, 10 out of 29 BD patients showed cerebellar symptoms as evidenced by the ICARS scale (see Table 1 for clinical details). Specifically, three patients were affected by standing balance and gait ataxia, three patients were affected by upper limb ataxia, and one patient was affected by dysmetric eye movements. In addition, 7 out of these 10 patients were also affected by fine postural hand tremor.

**Figure 1:**
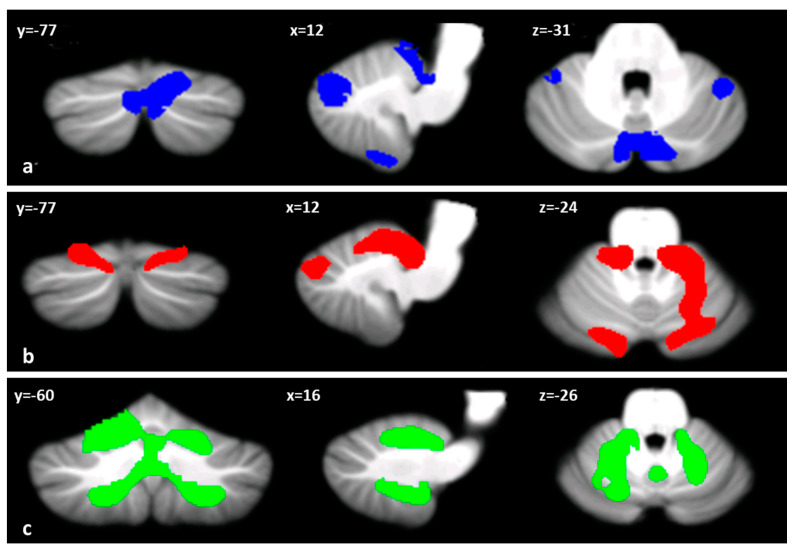
Cerebellar regions showing patterns of significantly reduced cerebellar grey matter (GM) are reported and superimposed on the spatially unbiased infratentorial template (SUIT) [51] in coronal (y), axial (z) and sagittal (x) sections. (**a**) BD < HS (in blue); (**b**) CD < HS (in red); (**c**) CD < BD (in green). The results are considered significant at *p*-values < 0.05 FWE corrected at the cluster level. Images are shown in the radiological convention.

**Figure 2:**
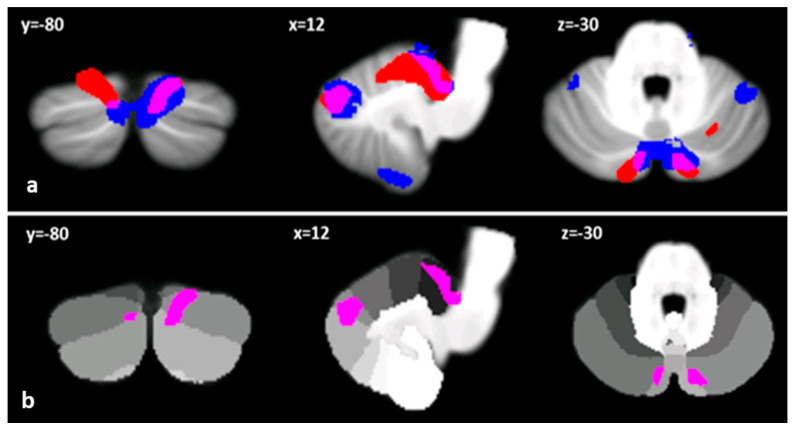
(**a**) Clusters of reduced cerebellar GM in BD and CD compared to HSs are reported in blue and red, respectively. Regions of overlapped cerebellar GM loss between BD and CD are shown in violet on the spatially unbiased infratentorial template (SUIT) [51]. (**b**) Only cerebellar regions showing patterns of significantly reduced GM in both BD and CD patients are shown in violet and superimposed on the probabilistic SUIT cerebellar atlas [51], which provides anatomical subdivision of cerebellar lobules (in greyscale). Images are shown according to radiological convention in coronal (y), axial (z) and sagittal (x) sections.

Voxel-wise analysis of cerebellar grey matter (GM) maps showed a significant pattern of GM loss in the cerebellar cortex of both BD and CD patients compared to the HS group (*p* = 0.05 family-wise error-FEW-corrected). More specifically, BD patients showed several clusters of significantly decreased GM volume that included right lobules I−IV and V, crus I, VIIB, IX and vermis crus II as well as left lobule VI and bilateral crus II (Figure 1a). When compared to HSs, CD patients also showed different clusters of cerebellar GM loss that diffusively affected both anterior and posterior cerebellar regions, including bilateral lobule VI, bilateral lobules I−IV, right V, bilateral crus I and Crus II (Figure 1b). Finally, the direct comparison between the two groups demonstrated a significant difference in GM loss in CD patients compared to BD patients, mainly involving anterior and posterior motor cerebellar regions such as bilateral lobules I−IV, V, VI, VIIIa and VIIIb, with extension in bilateral lobule IX and vermis VI (Figure 1c). Detailed statistics and peak voxels of voxel-based morphometry (VBM) analysis are reported in Table 2. Interestingly, the pattern of overlapping GM reduction in CD and BD patients was evident in the right lobule V, right crus I and bilateral crus II (Figure 2a,b).

## 3. Discussion

The aim of the present study was to clarify the potential role of the cerebellum in bipolar disorder by analysing the patterns of cerebellar alterations in BD patients compared to patients with neurodegenerative disorders selectively affecting the cerebellum. In this framework, the CD group was used as a model of cerebellar degeneration in order to highlight the common and distinct patterns of grey matter loss in comparison to BD in which cerebellar alterations have been detected [23].

The comparison between cerebellar-related neurodegenerative syndromes (such as SCA) and BD mainly arises from the growing evidence of a cerebellar involvement in manic symptoms of BD [49] and the presence of mood disturbance in cerebellar diseases [46]. As largely demonstrated, the posterior cerebellum is involved in the processing of cognitive and emotional information and takes part in the network involved in mentalizing and social interactions [16,40,41,49,52,53,54,55,56]. Indeed, the cerebellar cortex receives direct and indirect inputs from cortical associative areas and the midbrain, and in turn, the cerebellar nuclei send signals back to the limbic lobe and hypothalamic and thalamic nuclei, important relay stations that connect cortical and subcortical structures [19]. However, although cognitive and emotional cerebellar functions are widely described, the cerebellar role in bipolar disorder has been scarcely explored [22,23,30]. In light of these observations, our findings provide great insight into understanding the specific cerebellar contribution to the underlying neuropathophysiological mechanism of bipolar disorder.

Compared to controls, BD patients showed significant GM reduction in anterior cerebellar regions, including the right I−IV and V lobules; in posterior cerebellar areas, including right crus I and lobule IX; and in cerebellar vermis, including crus II, left lobule VI, and bilateral crus II.

According to the motor function of anterior cerebellar regions, the patterns of GM loss may be more related to the presence of clinical motor manifestations, i.e., psychomotor agitation [23,57], that, by definition, is influenced by the presence of the mania, and hypomania and mixed states of bipolar disorder throughout the progression of the disease (American Psychiatric Association. Diagnostic and Statistical Manual of Medical Disorders. 5th ed Washington, DC: American Psychiatric Association [58]). In light of these findings, it is reasonable to assume that the repeated mood relapses that characterize BD could have affected these cerebellar areas leading to structural modifications.

On the other hand, the extended pattern of GM reductions in hemispheric and vermal posterior cerebellar regions may be linked to the specific cognitive alterations described in BD studies in light of the cerebellar role in several emotional and cognitive domains [16,19,40,48,49,54,56,59], that characterize bipolar disorder [6,7,38,60,61,62,63].

It is worth noting that previous research reporting cerebellar alterations in bipolar disorder mainly used a whole-brain approach and did not focus on the cerebellum. In the present study, we implemented a procedure (see Methods section) that allowed us to restrict the analysis to the cerebellum and achieve anatomical localization of the specific cerebellar subregions involved [51]. Indeed, according to the cerebellar functional topography [19], we were able to identify the pattern of GM reduction in cerebellar portions, which have been linked to the cognitive, emotional and mood symptoms by several studies on bipolar patients [11,27,64,65,66,67,68].

When compared to controls, the CD group showed a diffuse pattern of GM loss throughout the cerebellar cortex. In line with the presence of typical cerebellar motor syndrome [19,55,69], an extensive pattern of GM loss involved motor anterior (i.e., I−IV, V) and posterior cerebellar regions (i.e., VIIIA and VIIIB). On the other hand, a pattern of GM loss was also found to extensively affect cognitive posterior cerebellar lobules, specifically crus I, crus II and lobe VI, in line with the presence of cognitive and emotional alterations as reported in CCAS [46,54,55,69,70,71,72]. Finally, when directly comparing BD and CD patients, significantly reduced cerebellar GM was found in the CD compared to the BD patients, only involving motor anterior cerebellar regions. This is in line with the presence of the cerebellar motor syndrome that is specific of CD patients and was not detected in our BD patients [19,55,69]. As previously stated, structural alterations in anterior cerebellar regions are also found in BD but they may be more related to the psychomotor agitation that typically accompanies the affective episodes. In spite of the presence of anterior cerebellar atrophy, CD patients did not show psychomotor agitation. This might mean that the involvement of the motor anterior cerebellar regions per se is not enough to evoke psychomotor agitation, which, by definition, is related to affective episodes that characterize bipolar patients and are subtended by a more complex dysfunctional network in which the cerebellum acts.

Interestingly, as depicted in Figure 2, BD and CD patients showed a common atrophy pattern specifically affecting right lobule V, right crus I and bilateral crus II, known to be typically involved in higher-order cognitive functions, whose alterations widely characterize both disorders [6,7,9,16,19,35,37,38,39,40,41,42,44,45,60,61,62,63].

Altogether, these results show for the first time common and different patterns of cerebellar alterations in BD and CD patients that may shed light on the potential cerebellar contribution to the neuropathophysiology of bipolar disorder. Specifically, according to these findings, it is possible to hypothesize that in BD patients, the pattern of cerebellar atrophy is specifically related to the cognitive dysfunctions and mood dysregulation typically reported in bipolar disorder. Indeed, the atrophy of posterior cerebellar lobules, in particular crus I and crus II, may be related the cognitive dysfunctions in line with the extensive connections with cortical association areas and, in particular, prefrontal cortical regions [19], while posterior vermal regions in the limbic cerebellum contribute to emotional lability and a flattening effect consistent with its extensive connections with the limbic system [73].

Interestingly, the specific involvement of the limbic cerebellum in mood regulation and behavior has been previously reported in a single-case study of a patient who showed behavioral abnormalities after the rupture of a cerebellar arteriovenous malformation [49]. Specifically, the behavioral symptoms were characterized by disinhibition or inappropriate behavior, emotional lability, irritability, aggressiveness, affective instability and impulsiveness together with the onset of a manic state. Data from MRI scans demonstrated an involvement of the posterior area of the cerebellar vermis, and cerebello-cerebral functional connectivity analysis revealed a pattern of altered connectivity in specific areas of the prefrontal-striatal-thalamic circuits that are typically altered in bipolar subjects during the manic state, suggesting a cerebellar role in mood regulation [49].

To conclude, there are some potential limitations that need to be discussed. With regard to the CD group and specifically to SCA2 sample, it has to be considered that extracerebellar signs may occur by definition on a subclinical level in SCA2 patients. However, as evidenced by the neurological examination and MRI analysis, the absence of extracerebellar signs and cortical atrophy has been ensured. Moreover, it must be taken into account that the choice of grouping cerebellar patients with different etiology depended on the rarity of this neurodegenerative condition that clearly affects the inclusion rate and makes it difficult to find large numbers of cerebellar patients with the same diagnosis. Furthermore, due to the retrospective nature of the study on CD patients, data on the presence of manic/mood symptoms were not available. Future studies may further address these issues and overcome these shortcomings. Another potential limitation is that all our BD patients were on medication, often involving polypharmacy, which is typical in patients with severe illness [74]. However, while different medication approaches can differently affect the central nervous system, it has to be considered that enrolling drug-free patients presents important ethical and clinical concerns.

Another issue that needs to be mentioned concerns the different profiles described in the literature (in terms of cognitive and mood symptoms) between the subtypes of BD patients. In the present study, the number of BD patients enrolled did not allow us to divide our sample into BD type 1 and type 2. Future studies that consider this different classification will aim to fill this gap, and to investigate the relationship between cerebellar structural changes and the severity of the BD symptoms providing further support for the present conclusions and opening new avenues for the therapeutic treatment of bipolar disorder specifically targeting the cerebellum.

## 4. Materials and Methods

### 4.1. Participants

Two groups of adult patients were enrolled in the study: subjects affected by BD and subjects affected by CDs.

BD patients were recruited from the bipolar disorder outpatient ward of the Department of Psychiatry, Policlinico Umberto I Hospital. All of the patients met the Diagnostic and Statistical Manual of Mental Disorders, Fifth Edition criteria [58] for BD, according to a diagnostic assessment performed with the Italian version of the Structured Clinical Interview for DSM-5—Clinician Version (SCID-5-CV) [75]. All patients had been euthymic for at least three months. The euthymic phase was established by using the Hamilton Depression Rating Scale (HDRS score < 10) [76] and Young Mania Rating Scale administration (YMRS score < 13) [77].

The inclusion criteria for BD patients were as follows: (i) aged between 18 and 60 years, (ii) euthymic mood for at least 3 months, (iii) first examination by a psychiatrist performed before age 40, and (iv) suitability for magnetic resonance imaging (MRI).

The exclusion criteria for BD patients were (i) having other Axis-I psychiatric disorders; (ii) exhibiting lifetime alcohol/substance abuse; (iii) having a history of an organic brain disorder or neurological disorder; (iv) having mental retardation; and (v) having a medical condition such as pregnancy, cardiovascular disease or diabetes. All BD patients were recruited by an expert clinical psychiatrist of the Department of Psychiatry, Policlinico Umberto I Hospital. Moreover, for each patient, the clinical diagnosis of BD in the euthymic phase was confirmed by another senior psychotherapist by means of a clinical interview, the HDRS [76] and YMRS [77].

Thirty-six patients with BD were initially included in the present study. Of the original group of patients, three refused to undergo the MRI exam, and four were excluded for the presence of moderate to severe brain vascular lesions (see exclusion criteria). Thus, the final sample was of 29 BD patients, and all participants underwent medical treatment (Table 3).

Thirty-two patients affected by CDs of different etiologies were also recruited (Table 4).

The inclusion criteria for CD patients were (i) more than 6 months of illness and (ii) evidence of diffuse cerebellar atrophy. The exclusion criteria for CD patients were (i) the presence of other pathological conditions (ii) the presence of any cortical lesion on conventional MRI scans and (iii) the presence of mental retardation.

Additionally, 37 sex- and age-matched HSs with no history of neurological or psychiatric illness were recruited as the control group.

All BD and CD patients underwent neurological evaluations. To quantify cerebellar motor deficits, the international cooperative ataxia rating scale [78], which ranges from 0 (absence of a motor deficit) to 100 (presence of motor deficits at the highest degree), was used.

In CD patients, the Wechsler adult intelligence scale—IV edition [79] was performed to exclude the presence of mental retardation.

Since these patients had already taken part in another study from our group, see Table 2 in Clausi and colleagues [40] for the cognitive profile.

This research study was approved by the Ethics Committee of Fondazione Santa Lucia IRCCS according to the principles expressed in the Declaration of Helsinki. Written informed consent was obtained from each subject. The main demographic and clinical characteristics are summarized in Table 1.

### 4.2. MRI Acquisition Protocol

Patients and HSs underwent MRI examination at 3T (Magnetom Allegra, Siemens, Erlangen, Germany) that included the following acquisitions: (1) dual-echo turbo spin echo [TSE] (TR = 6190 ms, TE = 12/109 ms); (2) fast-FLAIR (TR = 8170 ms, 204TE = 96 ms, TI = 2100 ms); and (3) 3D modified driven equilibrium Fourier transform (MDEFT) scans (TR = 1338 ms, TE = 2.4 ms, matrix = 256 × 224 × 176, in-plane FOV = 250 × 250 mm^2^, slice thickness = 1 mm) to perform voxel-based morphometry on cerebellar grey matter (GM) maps. To characterize the brain anatomy and determine the presence of macroscopic structural abnormalities, the TSE scans of patients were visually inspected by an expert neuro-radiologist. For HSs, conventional MRI scans were reviewed to ensure the absence of any macroscopic brain abnormality.

### 4.3. Image Processing and Analysis

The cerebellum was preprocessed individually using the SUIT toolbox [51] implemented in Statistical Parametric Mapping version 8 (Wellcome Department of Imaging Neuroscience; SPM-8 (http://www.fl.ion.ucl.ac.uk/spm/, accessed on 2 April 2009). The procedure was performed on individual T1 anatomical images and included isolating the cerebellum and then normalizing each cropped image into SUIT space and reslicing the probabilistic cerebellar atlas into individual subject spaces using the deformation parameters obtained by normalization. Finally, each segmented cerebellar GM map was smoothed using an 8-mm FWHM Gaussian kernel. Voxel-based morphometry was used to characterize the patterns of regional cerebellar GM atrophy in BD and CD patients compared to those of HSs. Additionally, a direct comparison between BD and CD patients was also carried out. Voxel-wise two-sample t-tests as implemented in SPM-8 were used. Sex was entered as a variable of no interest, and the analysis was restricted only to the voxels of the cerebellum by using an explicit exclusion mask. The results were considered significant at *p* values < 0.05 FWE corrected at the cluster level.

## 5. Conclusions

In conclusion, the present study demonstrates that BD and CD patients show a common pattern of altered cerebellar regions in lobules that are involved in cognitive and emotional abilities, such as crus I and crus II. Consistent with previous evidence, the alteration of these specific cerebellar portions may be the anatomical substrate that contributes to manic symptoms of bipolar disorders and the mood disturbances often observed in cerebellar diseases.

Altogether, these results provide useful insights for understanding and clarifying the cerebellar contribution to the pathophysiology of bipolar disorder and its potential role as a target for future treatments.

## Figures and Tables

**Table 1:** Main demographic and clinical characteristics of the BD, CD and HS groups.

Characteristic	BD (*n* = 29)	CD (*n* = 32)	HS (*n* = 37)
Age, years, mean ± SD	42.69 ± 10.53	46.81 ± 11.48	45.75 ± 14.26
Males/females	13/16	18/14	15/22
ICARS mean ± SD	1.10 ± 2.06	25.78 ± 12.94	−

SD = standard deviation; BD = bipolar disorder group; CD = cerebellar neurodegenerative disorders group; HS = healthy subjects group; ICARS = international cooperative ataxia rating scale.

**Table 2:** Cerebellar voxel-wise analyses between BD patients, CD patients and HSs. Detailed statistics and z-scores of peak voxels showing greatest statistical significance in the cluster. Cluster-forming threshold *p* < 0.05 FWE.

	Cluster Size (NoV)	Coordinates			Peak z-Score	Cerebellar Region
		x	z	y		
BD < HS	4667	7	−78	−33	5.35	R-Crus II
	3358	17	−42	−12	6.86	R-Lobule V
		20	−31	−19	5.95	R-Lobule V
	1024	45	−46	−27	4.36	R-Crus I
	892	−44	−47	−45	4.38	L-Crus II
	223	−41	−43	−28	4.37	L-lobule VI
CD < HS	17246	−8	−37	−19	5.99	L-Lobule I−IV
		12	−37	−22	5.75	R-Lobule I−IV
		13	−48	−16	5.21	R-Lobule V
	2047	−12	−87	−29	4.77	L-Crus II
		−18	−79	−21	4.65	L-Crus I
CD < BD	28567	22	−55	−45	6.78	R-Lobule VIIIb
		−20	−61	−48	5.53	L-Lobule VIIIa
		−14	−57	−19	5.52	L-Lobule VI

NoV = number of voxels; R = right; L = left.

**Table 3:** Clinical details regarding the BD group.

Medical Treatment	Mean ± SD	N°
HDRS	1.82 ± 2.68	29
YMRS	1.61 ± 3.01	29
Current pharmacotherapy		
Antipsychotics		13
Lithium		14
Antiepileptics		22
Antidepressants		2
Anxiolytic		3
Polypharmacy		17

HDRS = Hamilton Depression Rating Scale; YMRS = Young Mania Rating Scale; SD = standard deviation.

**Table 4:** Clinical details regarding the CD group.

Diagnosis	N°
SCA type1	1
SCA type 2	12
SCA type 6	1
SCA type 15	1
SCA type 28	1
SPG7	6
FRDA	2
ICA	8

SCA = spinocerebellar ataxia; SPG7 = spastic paraplegia type 7; FRDA = Friedreich’s ataxia; ICA = idiopathic cerebellar atrophy.

## Data Availability

Study data will be shared upon request.
